# Competitive Inhibition of Thyroidal Uptake of Dietary Iodide by Perchlorate Does Not Describe Perturbations in Rat Serum Total T_4_ and TSH

**DOI:** 10.1289/ehp.0800111

**Published:** 2009-01-05

**Authors:** Eva D. McLanahan, Melvin E. Andersen, Jerry L. Campbell, Jeffrey W. Fisher

**Affiliations:** 1 University of Georgia, Department of Environmental Health Sciences, Athens, Georgia, USA;; 2 Hamner Institutes for Health Sciences, Division of Computational Biology, Research Triangle Park, North Carolina, USA

**Keywords:** BBDR model, HPT axis, iodide, mode of action, PBPK model, perchlorate, rat, sodium/iodide symporter, thyroid, thyroid hormone secretion

## Abstract

**Background:**

Perchlorate (ClO_4_^−^) is an environmental contaminant known to disrupt the thyroid axis of many terrestrial and aquatic species. ClO_4_^−^ competitively inhibits iodide uptake into the thyroid at the sodium/iodide symporter and disrupts hypothalamic–pituitary–thyroid (HPT) axis homeostasis in rodents.

**Objective:**

We evaluated the proposed mode of action for ClO_4_^−^-induced rat HPT axis perturbations using a biologically based dose–response (BBDR) model of the HPT axis coupled with a physiologically based pharmacokinetic model of ClO_4_^−^.

**Methods:**

We configured a BBDR-HPT/ClO_4_^−^ model to describe competitive inhibition of thyroidal uptake of dietary iodide by ClO_4_^−^ and used it to simulate published adult rat drinking water studies. We compared model-predicted serum thyroid-stimulating hormone (TSH) and total thyroxine (TT_4_) concentrations with experimental observations reported in these ClO_4_^−^ drinking water studies.

**Results:**

The BBDR-HPT/ClO_4_^−^ model failed to predict the ClO_4_^−^-induced onset of disturbances in the HPT axis. Using ClO_4_^−^ inhibition of dietary iodide uptake into the thyroid, the model underpredicted both the rapid decrease in serum TT_4_ concentrations and the rise in serum TSH concentrations.

**Conclusions:**

Assuming only competitive inhibition of thyroidal uptake of dietary iodide, BBDR-HPT/ClO_4_^−^ model calculations were inconsistent with the rapid decrease in serum TT_4_ and the corresponding increase in serum TSH. Availability of bound iodide in the thyroid gland governed the rate of hormone secretion from the thyroid. ClO_4_^−^ is translocated into the thyroid gland, where it may act directly or indirectly on thyroid hormone synthesis/secretion in the rat. The rate of decline in serum TT_4_ in these studies after 1 day of treatment with ClO_4_^−^ appeared consistent with a reduction in thyroid hormone production/secretion. This research demonstrates the utility of a biologically based model to evaluate a proposed mode of action for ClO_4_^−^ in a complex biological process.

Perchlorate (ClO_4_^−^), a water-soluble chemical distributed extensively in the environment, has caused public health concerns because of widespread exposures to populations by ingestion of ClO_4_^−^-contaminated food and water. The ClO_4_^−^ anion has recently been classified as a ubiquitous environmental contaminant throughout the United States, with detectable concentrations in many drinking water supplies (Motzer 2001) and food and beverage products (e.g., milk, lettuce, grains) (El Aribi et al. 2006), and is also found as a contaminant in dietary supplements (Snyder et al. 2006). The occurrence of ClO_4_^−^ in the environment is most often attributed to anthropogenic uses of ClO_4_^−^ salts as oxidizers in solid rocket propellants and application of Chilean nitrate fertilizers (Motzer 2001); however, ClO_4_^−^ is also formed naturally by atmospheric processes (Dasgupta et al. 2005). The primary health concern for environmental exposures to ClO_4_^−^ is adverse (and irreversible) neurodevelopmental outcomes mediated by disruption of the hypothalamic–pituitary–thyroid (HPT) axis in the immature fetus or child. Adequate levels of thyroid hormones are essential for proper growth, development, reproduction, and metabolism.

ClO_4_^−^ blocks thyroidal uptake of radiolabeled iodide and alters thyroid hormone homeostasis [an increase in serum thyroid-stimulating hormone (TSH) concentrations and a decline in serum thyroxine (T_4_) concentrations] (National Research Council 2005; U.S. Environmental Protection Agency 2002). Dietary iodide is an essential nutrient used in the formation of thyroid hormones, and if in short supply, from the blocking effect of ClO_4_^−^, an iodide-deficiency–induced decrease in thyroid hormone production is thought to occur. For humans, under clinical conditions, ClO_4_^−^ clearly inhibits thyroidal uptake of radiolabeled iodide (Greer et al. 2002); however, disruption of the HPT axis by ClO_4_^−^ remains to be clearly demonstrated in euthyroid adults. Interestingly, ClO_4_^−^ was used to treat thyrotoxicosis (overactive thyroid gland) with large doses, ranging from 17–29 mg/kg, until several cases of fatal aplastic anemia occurred in the early 1960s. The mechanism of action for this adverse effect of ClO_4_^−^ is unknown. ClO_4_^−^ was also used clinically to test thyroid function, referred to as the ClO_4_^−^ discharge test. For this test, radiolabeled tracer iodide is administered a few hours before dosing with 2–13 mg/kg ClO_4_^−^ (Wolff 1998), causing blocking of active uptake of radiolabeled iodide for several hours. By 2 hr after dosing, the thyroid gland has sequestered radiolabeled iodide, but circulating levels of the iodide remain in the body and are available for sequestration into the thyroid gland. If the thyroid gland is defective in binding (trapping) iodide, rapid and excessive efflux of radiolabeled iodide from the thyroid is observed in the presence of ClO_4_^−^.

The primary mechanism by which ClO_4_^−^ blocks thyroidal uptake of iodide is competitive inhibition at the sodium/iodide symporter (NIS) protein. The NIS protein is responsible for active translocation of both iodide and ClO_4_^−^ from circulating blood to the thyroid follicle. Bidirectional passive diffusion of iodide and ClO_4_^−^ also occurs between the thyroid gland and the blood supply perfusing the thyroid. When the ClO_4_^−^ discharge test is administered, a possible mechanism of action for efflux of radiolabeled iodide from defective thyroid glands may be simple diffusion of radiolabeled iodide down a concentration gradient from the thyroid gland into the blood supply because the NIS “pump” that maintains an iodide gradient between the blood and the thyroid gland is shut down by ClO_4_^−^. A rapid efflux of radiolabeled iodide has been observed in cell preparations deficient in the binding protein, thyroglobulin (Kosugi et al. 1996).

Several decades ago, *in vitro* studies were conducted in Sprague-Dawley rat thyroid homogenates to examine the effects of antithyroid compounds on iodide peroxidase activity (Alexander 1959). For each antithyroid compound tested, thyroid homogenates were incubated with 1 μmol potassium iodide (KI) and 2 × 10^−3^ M antithyroid compound for 2 hr. Thiocyanate, thiouracil, and cyanide were several of the antithyroid compounds shown to inhibit incorporation of ^131^I into L-tyrosine, decreasing the formation of monoiodotyrosine (MIT) and diiodotyrosine (DIT). However, ClO_4_^−^ did not significantly alter the synthesis of MIT and DIT. Thus, the data suggest that ClO_4_^−^ does not alter organification of iodide, which is a key step in thyroid hormone synthesis (Alexander 1959). Several years later, Greer et al. (1966) provided contradicting results, demonstrating a direct effect of ClO_4_^−^ on the rat thyroid gland *in vitro*. These authors reported that ClO_4_^−^ reduced formation of MIT and DIT in rat thyroid lobes when incubated with ClO_4_^−^. They observed changes in MIT and DIT formation for ClO_4_^−^ media concentrations starting at 10 mg/L, with a reported 50% effective reduction of DIT at 250 mg/L.

In addition to ClO_4_^−^ acting at the NIS protein of the thyroid gland, a few other studies have demonstrated another potential mode of action (MOA) for high doses of ClO_4_^−^ to disrupt the HPT axis. Using an *in vitro* system and very high concentrations of ClO_4_^−^ (≥ 1,700 mg/L), Okabe and Hokaze (1993) reported that ClO_4_^−^ displaces T_4_ bound to bovine serum albumin. This observation implies that free T_4_ (fT_4_) dislodged from serum proteins would then be cleared from the body more quickly, leading to a disruption of the HPT axis. In earlier *in vivo* studies, Yamada (1967), who was interested in this same MOA, removed the thyroid or gave methimazole [methylmercaptoimidazole (MMI)] to rats to inhibit thyroid function (and treated them with T_4_) and then evaluated the ClO_4_^−^ dose-dependent decline in serum protein-bound iodide (PBI; primarily radiolabeled T_4_). The decline in serum PBI was interpreted as displacement of protein bound T_4_ by ClO_4_^−^. The daily intake rates of ClO_4_^−^ were estimated to be 10–1,000 mg/kg/day.

Historically, pharmacokinetic or computational analyses of the HPT axis have played a prominent role in efforts to quantify and understand the complex relationships between biological action of thyroid hormones and their production, metabolism, transport, distribution, and interaction with receptors (DiStefano and Landaw 1984; Oppenheimer 1983). Recently, physiologically based pharmacokinetic (PBPK) models were developed to describe the kinetics of ClO_4_^−^ and radiolabeled iodide and the interaction of ClO_4_^−^ on the thyroidal uptake of radiolabeled iodide in the adult rat (Fisher et al. 2000; Merrill et al. 2003). The rodent PBPK model described transport of ClO_4_^−^ into the thyroid gland by the NIS protein because high concentrations of ClO_4_^−^ were measured in thyroid tissue relative to serum (Yu et al. 2002). High concentrations of ClO_4_^−^ in the thyroid gland had also been observed in laboratory animals administered ClO_4_^−^ (Chow et al. 1969; Chow and Woodbury 1970).

However, using an indirect electrochemical technique to infer movement of ClO_4_^−^ by the NIS into thyroid cells, Riedel et al. (2001) concluded that ClO_4_^−^ was not taken up into the thyroid gland because an electrical gradient was not created. Evidence for ClO_4_^−^ movement into the thyroid gland was reviewed by Wolff (1998) and Clewell et al. (2004). Finally, a recent study directly measured the TSH-mediated movement of ClO_4_^−^ into NIS-expressing FRTL-5 rat thyroid cells. This research provided conclusive evidence that the NIS protein did translocate ClO_4_^−^ into the thyroid cells (Tran et al. 2008). At the same time, Dohan et al. (2007) concluded that ClO_4_^−^ is transferred by the NIS based on indirect lines of evidence using another anion (perrhenate) that is also transported with an electroneutral stoichiometry into thyroid cells.

In the study we report here, using computational analysis, we tested the hypothesis that the primary MOA of ClO_4_^−^ on the HPT axis is competitive inhibition of uptake of thyroidal iodide. We combined our recently published biologically based dose–response (BBDR) model for the HPT axis (McLanahan et al. 2008), which we calibrated to describe perturbations resulting from dietary iodide deficiency, with a simple PBPK model for ClO_4_^−^. The published ClO_4_^−^ dose–response data sets for the adult rat HPT axis (Männistö et al. 1979; Yu et al. 2002) were simulated with our combined BBDR-HPT/ClO_4_^−^ models. Additionally, we conducted limited *in vitro* experiments at relevant ClO_4_^−^ plasma concentrations to evaluate the role of ClO_4_^−^ in displacement of T_4_ from serum-binding proteins.

## Materials and Methods

### Laboratory experiments

#### Displacement of T_4_ from serum proteins

Radiolabeled *L*-[3′,5′-^125^I]thyroxine (^125^I-T_4_; specific activity, 1,500 μCi/μg; Perkin Elmer Life Sciences, Waltham, MA) was prepared by diluting 25 μCi (0.5 mL of 50 μCi/mL solution) with 1.0 mL 10% bovine serum albumin solution and predialyzing it against 2 mL 0.15 M phosphate buffer solution to reduce the amount of free ^125^I in the solution. We spiked male rat and male human serum (both obtained from Bioreclamation Inc., Hicksville, NY) with ClO_4_^−^ (Sigma Aldrich, Milwaukee, WI; 0.01 mL ClO_4_^−^/mL serum) diluted in saline to give a serum concentration of 0, 1.0, 10.0, 50.0, 100, 200, or 300 μg/mL, vortexed the solution, and allowed it to stand for 30 min. We then added 0.02 mL (200,000–300,000 cpm) predialyzed ^125^I-T_4_ solution/mL serum, vortexed the solution, and allowed it to stand for 30 min. One mL of spiked serum was dialyzed across a 6,000-kDa membrane overnight against 1.0 mL 0.15 M phosphate buffer (pH 7.3) at 37°C in a gently shaking water bath. Serum and dialysate were removed from each half-cell with a Pasteur pipette precoated with carrier solution. We immediately placed 750 μL dialysate into 750 μL carrier solution (3.0 mg/mL T_4_ and 3.72 mg/mL NaI in 0.5 M NaOH) and vortexed it. Volume recovered was assessed in order to account for possible fluid shifts across the membrane. The dialysate/carrier solution was further processed by adding 1.5 mL magnesium precipitating solution (6.05 g/L Trizma base, 5.85 g/L NaCl, and 100 g/L MgCl_2_·6H_2_O, pH 9.274); the solution was vortexed and allowed to stand for 10 min. The precipitate was centrifuged for 5 min at 2,000 rpm and the supernatant was removed. The pellet was washed three times by resuspending it in 1.5 mL washing solution (precipitate solution with pH adjusted to 8.772), centrifuging it at 2,000 rpm for 5 min, and removing the supernatant. We assessed the tube with 750 μL serum and the pellet precipitated from 750 μL dialysate by gamma counting and compared the results with control incubations.

### Model development

#### HPT axis

**Figure 1:**
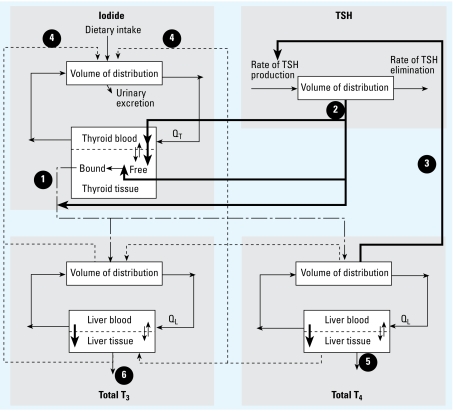
BBDR model structure of the HPT axis for the description of dietary iodide, TSH, TT_4_, and TT_3_. The model provides for recycling of dietary iodide released from metabolism of thyroid hormones (dashed lines), as well as the stimulation and regulation of the HPT axis by TSH. Numbers indicate several key processes: 1, loss of thyroidal-bound iodide secreted as thyroid hormones; 2, TSH stimulation of NIS iodide uptake, organification of iodide, and stimulation of thyroid hormone production; 3, T_4_ negative feedback on TSH production (thick solid lines); 4, formation of free iodide from serum and liver T_3_ and T_4_ metabolism; 5, phase II metabolism of T_4_ and excretion into feces; and 6, fecal elimination of T_3_. For additional details on the BBDR-HPT axis model, see McLanahan et al. (2008). Bold arrows represent active transport and double thin arrows represent passive diffusion processes. Thin solid lines represent blood flows to or from the thyroid (Q_T_) or the liver (Q_L_).

We constructed the BBDR-HPT axis model in acslXtreme, version 2.4 (AEgis Technologies, Huntsville, AL) and solved it using the Gear algorithm for stiff systems. The model for dietary iodide and the thyroid axis was used as previously described (McLanahan et al. 2008). Briefly, this BBDR-HPT axis model includes submodels for dietary iodide, TSH, and the thyroid hormones T_4_ and T_3_ (Figure 1). The submodels combine to form a simplified, quantitative description of the thyroid axis in the adult rat. Regulatory and compensatory effects of TSH were empirically described for several thyroidal processes, including TSH stimulation of *a*) NIS thyroidal iodide uptake, *b*) formation of thyroid hormone precursors, and *c*) thyroid hormone secretion. The TSH/T_4_ negative feedback loop is described using the relationship of serum total T_4_ (TT_4_) and TSH, as well as the metabolism of thyroid hormones with recycling of iodide.

**Figure 2:**
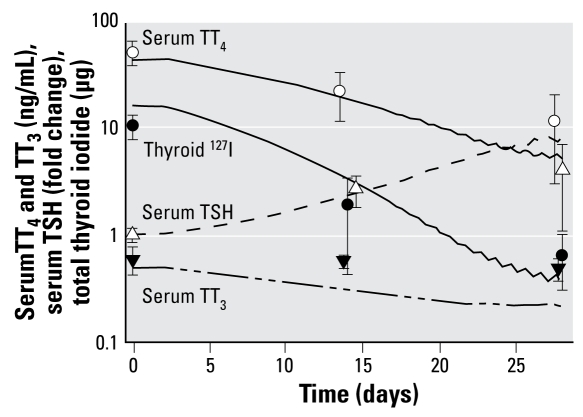
BBDR-HPT axis model simulation (lines) of HPT axis alterations in serum TT_4_, TT_3_, and TSH and thyroid iodide up to 28 days after administration of a low-iodide diet (0.33 μg/day). Data (mean ± SD) are adapted from Okamura et al. (1981) and are offset at days 14 and 28 to aid in visualization of data.

We developed the BBDR-HPT axis model (McLanahan et al. 2008) to predict perturbations in serum TT_4_, total T_3_ (TT_3_), TSH, and total thyroidal iodide content that resulted from a decrease in dietary iodide intake. Normal laboratory rat iodide intake is approximately 20 μg/day. Figure 2 shows a model simulation up to 28 days for Simonsen Albino rats placed on a diet containing 0.33 μg iodide per day from day 0 through day 28 (Okamura et al. 1981).

#### Perchlorate

**Figure 3:**
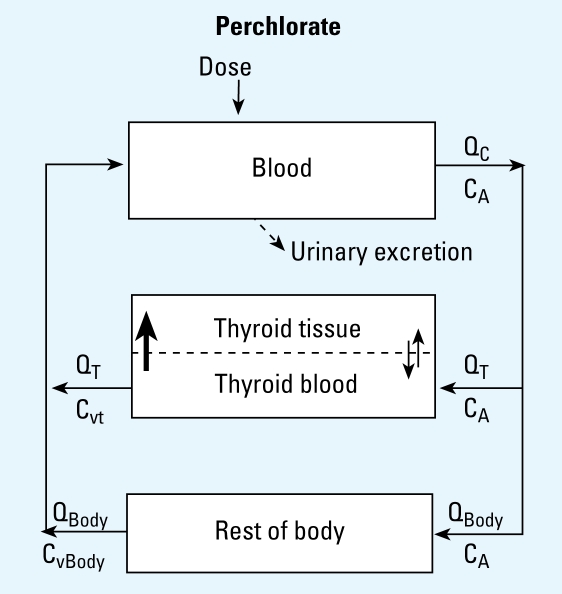
ClO_4_^−^ PBPK model structure for the adult male rat. ClO_4_^−^ (intravenous or oral drinking water dose) enters the plasma (Dose), where it is distributed to tissues or excreted in urine. Thyroid is modeled as diffusion limited, with active uptake (bold arrow) into the thyroid via the NIS. The “Rest of Body” compartment is a flow-limited compartment and includes all other body tissues in which ClO_4_^−^ may distribute. Double thin arrows represent passive diffusion. Abbreviations: C_A_, concentration of ClO_4_^−^ in arterial blood; C_vBody_, concentration of ClO_4_^−^ in venous blood leaving the rest of the body compartment; C_vt_, concentration of ClO_4_^−^ in venous blood leaving thyroid; Q_C_, cardiac output; Q_Body_, blood flow to the rest of the body; Q_T_, blood flow to the thyroid.

Instead of the more elaborate modeling approach implemented by Merrill et al. (2003), a simple model structure for ClO_4_^−^ was constructed (Figure 3), similar to the approaches reported by Crump and Gibbs (2005) and Lorber (2008). This PBPK model consisted of three compartments: plasma, thyroid, and the rest of the body. ClO_4_^−^ is rapidly absorbed after oral administration and is distributed throughout the body but excreted unchanged through the urine (Wolff 1998). Urinary excretion of ClO_4_^−^ is described by first-order clearance from the plasma. The thyroid gland is described with a blood and tissue compartment using diffusion limitation and active uptake into the thyroid via the NIS. Several studies have shown that ClO_4_^−^ and ^36^ClO_4_^−^ are transported into the thyroid via the NIS, and an increased uptake has been observed in rats administered TSH and ClO_4_^−^ (Anbar et al. 1959; Chow et al. 1969; Chow and Woodbury 1970; Dohan et al. 2007; Goldman and Stanbury 1973; Tran et al. 2008; Yu et al. 2002).

### BBDR-HPT axis and ClO_4_^−^ PBPK model integration

We linked the PBPK model for ClO_4_^−^ with the BBDR-HPT axis model (McLanahan et al. 2008) by competition between ClO_4_^−^ and iodide at the NIS protein. Equations 1 and 2 describe, respectively, the competitive interaction between ClO_4_^−^ and the NIS protein and TSH stimulation of NIS activity:


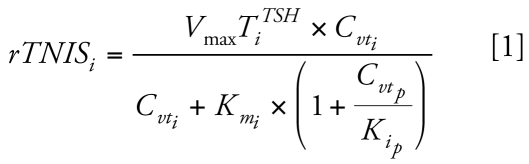



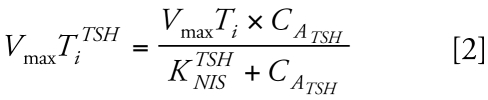


where *rTNIS_i_* is the rate of iodide actively transported into the thyroid via NIS (nanomoles per hour), *C_vt_*_*_i_*_ is the free concentration of iodide in thyroid blood (nanomoles per liter), *K_m_*_*_i_*_ is the affinity constant of iodide for the NIS (nanomoles per liter), *C_vt_*_*_p_*_ is the concentration of ClO_4_^−^ in the thyroid blood (nanomoles per liter), *K_i_*_*_p_*_ is the inhibition constant of ClO_4_^−^ for NIS iodide transport (nanomoles per liter), *V*_max_ *T_i_* is the maximum rate of NIS iodide uptake (nanomoles per hour), *C_A_*_*_TSH_*_ is the serum concentration of TSH (nanomoles per liter), and *K_NIS_^TSH^* is the concentration of TSH that gives rise to half-maximal rate of NIS transport of iodide (nanomoles per liter). We previously simultaneously optimized *V*_max_ *T_i_* (nanomoles per hour) and *K_NIS_^TSH^* (nanomoles per liter) (McLanahan et al. 2008) to euthyroid, dietary-iodine–sufficient (~ 20 μg/day) rat data for serum and thyroid iodide reported by Eng et al. (1999) and McLanahan et al. (2007), respectively. The other parameter, NIS affinity constant for iodide (*K_m_*_*_i_*_, nanomoles per liter) in Equation 1 was obtained from Gluzman and Niepomniszcze (1983) as reported by McLanahan et al. (2008).

We used an equation similar to Equation 1 to model iodide’s inhibition of the uptake of ClO_4_^−^ via the NIS; however, the effect of iodide on transport of ClO_4_^−^ into the thyroid is minimal because ClO_4_^−^ has a higher affinity for the NIS protein compared with iodide (1,500 nmol ClO_4_^−^/L vs. 31,519 nmol I/L).

#### Model parameter values

We obtained physiologic parameters (Table 1) for tissue volumes and blood flows from previous studies (Brown et al. 1997; Everett et al. 1956; Malendowicz and Bednarek 1986; McLanahan et al. 2007, 2008). The BBDR–HPT axis model parameters had been calibrated for the euthyroid adult rat previously and used to describe iodide-deficient conditions (McLanahan et al. 2008).

Table 2 shows model parameters for ClO_4_^−^. We set the affinity constant, *K_i_*_*_p_*_, for the inhibition of iodide uptake by ClO_4_^−^ equal to *Km_p_*, the measured affinity constant for ClO_4_^−^ and the NIS. A *K_m_*_*_p_*_ value of 1.5 μM was used, which was reported as an inhibition affinity constant for uptake of radiolabeled iodide in thyroid cells (Kosugi et al. 1996). We calculated a ClO_4_^−^ tissue:blood partition coefficient for the body compartment (minus the thyroid and plasma) by weighting the partition coefficients based on tissue volume for tissue:blood partition coefficient values reported in Merrill et al. (2003) for the adult male rat. Optimization was carried out using acslXtreme parameter estimation tool kit, using ^36^ClO_4_^−^ kinetic data reported by Yu et al. (2002), who administered a single intravenous bolus dose of 3.3 mg/kg ^36^ClO_4_^−^. Optimized values were obtained for *V*_max_ *Tc*_*_p_*_, the maximal rate of uptake of ^36^ClO_4_^−^ into the thyroid and urinary clearance constant, and for *ClU_c_*_*_p_*_, a term used to describe the rate of excretion of ^36^ClO_4_^−^ in urine. The *ClU_c_*_*_p_*_ value was obtained by fitting both the urinary excretion of ^36^ClO_4_^−^ and serum concentrations of ^36^ClO_4_^−^.

### Experimental simulations

Yu et al. (2002) administered 0, 0.1, 1, 3, and 10 mg ClO_4_^−^/kg/day in drinking water to adult male Sprague-Dawley rats and determined serum TT_4_, fT_4_, TT_3_, TSH, and ClO_4_^−^ concentrations after exposures of 1, 5, and 14 days. We did not consider the Yu et al. (2002) data for fT_4_ for this study because the increase in fT_4_ with ClO_4_^−^ concentration is considered to be erroneous owing to a nondialysis method. The problem with this method has been previously discussed (Fisher et al. 2006). In addition, Männistö et al. (1979) administered ClO_4_^−^ at a rate of 15 mg/kg/day in drinking water to adult male Sprague-Dawley rats and measured serum TT_4_, TT_3_, and TSH after exposure for 0, 2, 4, 6, 9, and 14 days. The BBDR-HPT/ClO_4_^−^ model was configured to simulate these experiments. Ingestion of ClO_4_^−^ in drinking water took place over a 12-hr period.

## Results

### Laboratory binding and displacement experiments

ClO_4_^−^ had little or no effect on displacing ^125^I-T_4_ from serum proteins for both human and rats [see Supplemental Material, Figure 1 (http://www.ehponline.org/members/2009/0800111/suppl.pdf)] across the relevant range of ClO_4_^−^ concentrations tested (1–300 μg/mL). The highest concentration tested, 300 μg/mL, is 30 times greater than the range of interest for ClO_4_. Thus, older studies that examined high doses of ClO_4_^−^ probably caused a displacement in protein-bound T_4_, but for the concentration range of interest, 0.01–1.0 μg/mL (corresponding to a ClO_4_^−^ dose of 0.01–10 mg/kg/day), there appears to be little displacement of protein-bound T_4_ by ClO_4_^−^. We found no significant differences by analysis of variance for either rat (*p* = 0.13) or human (*p* = 0.96) serum. Therefore, this MOA was not explored in the computational analysis.

### PBPK model predictions

**Figure 4:**
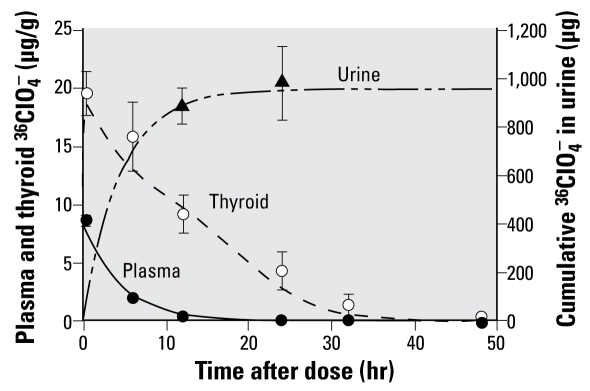
Serum and thyroid concentration and cumulative urinary excretion of ^36^ClO_4_^−^ after a 3.3-mg/kg intravenous dose. Lines represent model predictions. Data (mean ± SD) are from Yu et al. (2002).

Figure 4 shows model-predicted and laboratory-observed serum and thyroid ^36^ClO_4_^−^ concentrations and cumulative urinary excretion of ^36^ClO_4_^−^ after a single intravenous bolus dose of 3.3 mg/kg ^36^ClO_4_^−^. We obtained these successful predictions of thyroidal and serum concentrations of ^36^ClO_4_^−^ and urinary clearance using an optimized *V*_max_ *T_c_*_*_p_*_ value of 1,800 nmol/hr/kg^0.75^ and an optimized *ClU_c_*_*_p_*_ value of 0.007 L/hr/kg^0.25^ (Table 2). Simulations of ClO_4_^−^ in serum and the thyroid gland were similar to previous model findings of Merrill et al. (2003) for the drinking water dose groups of 0.1, 1.0, 3.0, and 10 mg/kg/day that were originally reported by Yu et al. (2002) (data not shown).

### HPT response predictions: testing the hypothesis

**Figure 5:**
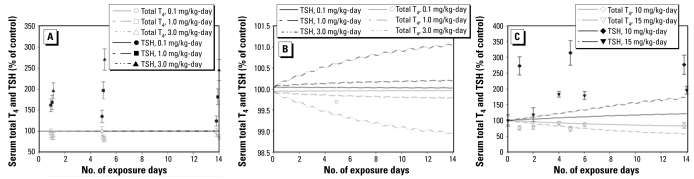
(*A*) Model predictions (lines) of serum T_4_ and TSH after exposure to 0.1, 1, or 3 mg/kg/day ClO_4_^−^ in drinking water. Data for TSH and T_4_ from Yu et al. (2002) (0.1, 1.0, 3.0 mg/kg/day). (*B*) A closer look at model simulations in *A*, expanding the *y*-axis. (*C*) Model predictions of serum T_4_ and TSH after exposure to 10 or 15 mg/kg/day ClO_4_^−^ in drinking water. Data (mean ± SD) for T_4_ and TSH for 10 mg/kg/day (~ 350 g rat) from Yu et al. (2002), and for 15 mg/kg/day (~ 200 g rat) from Männistö et al. (1979). The model does not predict the rapid changes in serum T_4_ and TSH observed in these studies.

We linked the ClO_4_^−^ model with the BBDR-HPT axis model that we previously calibrated to predict serum TT_4_, TT_3_, TSH, and total thyroid iodide for sufficient and insufficient dietary iodide intakes and evaluated the ability of the combined model to predict ClO_4_^−^-induced HPT axis disturbances. The hypothesis was that the BBDR-HPT/ClO_4_^−^ model would predict the HPT axis disturbances based on competitive inhibition of thyroidal uptake of dietary iodide (Equation 1) for ClO_4_^−^ drinking water dose rates reported by Männistö et al. (1979) (15 mg/kg/day) and Yu et al. (2002) (0.1, 1, 3, 10 mg/kg/day). In this case, the BBDR-HPT/ClO_4_^−^ model failed to predict the HPT axis responses. Dose-dependent increases in serum TSH concentrations were severely underpredicted, and dose-dependent decreases in serum TT_4_ concentrations were also under-predicted (Figure 5A,B). The small decrease in serum TT_3_ in response to 10 mg/kg/day ClO_4_^−^ was better predicted by the model [see Supplemental Material, Figure 2 (http://www.ehponline.org/members/2009/0800111/suppl.pdf)].

Yu et al. (2002) reported dose-dependent serum TSH increases ranging from 60% for the lowest dose to 174% for the highest dose compared with controls after 1 day of ClO_4_^−^ treatment. For all dose groups, serum TSH level continued to increase by day 5 of treatment, with the exception of the 0.1 mg/kg/day dose group. By day 14 of treatment, TSH concentrations were in decline relative to the peak measured TSH concentrations. Männistö et al. (1979) reported that 15 mg/kg/day caused serum TSH levels to increase by 18% on day 2 of treatment and by 96% on day 14 (Figure 5C). These measured TSH concentrations are in stark contrast to the model-predicted values (Figure 5C). The model-simulated serum TSH concentrations were predicted to increase only by 2–6% after 1 day of treatment and by 21–36% after 14 days of treatment with the highest ClO_4_^−^ doses (10 and 15 mg/kg/day; Figure 5C).

Similar to the TSH findings, the dose-dependent decline in serum TT_4_ concentrations reported by Yu et al. (2002) ranged from 6% for the lowest dose to 24% for the highest dose after 1 day of ClO_4_^−^ treatment. Consistent with the increases in serum TSH on day 5, all treatment groups, except for the 0.1 mg/kg/day ClO_4_^−^ dose group, displayed a continued decline in serum TT_4_ concentrations. By day 14 of ClO_4_^−^ treatment, TT_4_ levels were recovering (Figure 5A). Model predictions of serum TT_4_ concentrations were also in disagreement with observations. The model-predicted decreases in serum TT_4_ concentrations were only 2–6% by day 1 of treatment and 13–26% by day 14 for the 10- and 15-mg/kg/day ClO_4_^−^ dose groups. Although the model-predicted and observed serum TT_4_ concentrations were in agreement for the 10-mg/kg/day group by day 14, the temporal aspect of the TT_4_ perturbations was not predicted (Figure 5C).

The model-predicted interaction of ClO_4_^−^ (competitive inhibition) on thyroidal uptake of dietary iodide (Table 3) demonstrated the nonlinear uptake behavior of dietary iodide that is described by Michaelis-Menten kinetics for each anion. NIS-mediated thyroidal uptake of dietary iodide (micrograms per day) was predicted to be similar on days 1 and 14 (Table 3), with a slight increase on day 14 because of TSH stimulation of the NIS for the two highest ClO_4_^−^ doses (10 and 15 mg/kg/day). Although thyroidal iodide stores were not reported by Yu et al. (2002) and Männistö et al. (1979), only a 6% and 12% depletion was predicted to occur after 1 day and 2 weeks of treatment of ClO_4_^−^ for dose rates of 10 and 15 mg/kg/day, respectively (Table 3). The model-predicted depletion of thyroidal iodide stores was 33% and 46% by day 14 for these two doses, respectively.

The model-predicted percent inhibition in thyroidal uptake of dietary iodide was predicted to range from 26% for the 0.1 mg/kg ClO_4_^−^ dose group to 96% and 97% inhibition for the two highest dose groups (Table 3).

#### Is ClO_4_^−^ altering thyroid hormone secretion?

The best approach to answer this question would be to design studies focused on evaluating the status of the thyroid gland for a known intake of dietary iodide, such as measuring the ratios of MIT and DIT, and thyroid hormones in animals treated with ClO_4_^−^. Another equally important and time-intensive effort would be to develop a mechanistic-based model of the thyroid gland that would include explicit descriptions of the synthesis and secretion pathways for thyroid hormones, for which some data are still lacking. Instead, we simply imposed “non-mechanistic” conditions on the thyroid gland by limiting thyroid hormone production and asking whether the systemic concentrations of TT_4_ and TSH can be described by simply limiting thyroid hormone production and secretion. To account for inhibition of thyroid hormone production, we modified equation 14 from McLanahan et al. (2008), which describes the overall rate of thyroid hormone production (*rTH_prod_*, nanomoles per hour), by the addition of a proportional inhibition term (*INH_p_*), such that


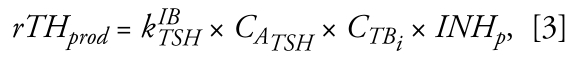


where *k_TSH_^IB^* (square liters/nanomole per hour) is a linear rate term, *C_A_*_*_TSH_*_ (nanomoles per liter) is the concentration of TSH in the serum, and *C_TB_*_*_i_*_ (nanomoles per liter) is the bound concentration of iodide in the thyroid as thyroid hormone precursors. The production rate of T_4_ and T_3_ was described as a fraction of the total production rate, and the proportion of T_4_ and T_3_ changes as a result of depletion of thyroidal iodide stores (McLanahan et al. 2008).

**Figure 6:**
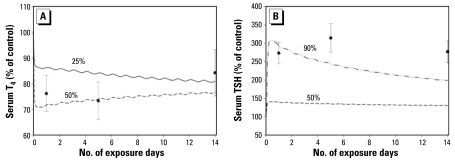
Serum T_4_ model predictions (*A*; lines) and serum TSH model predictions (*B*; lines) shown as percentage of control (100%) values at a ClO_4_^−^ dose of 10 mg/kg/day and varying degrees of inhibition of T_4_ and T_3_ synthesis and secretion from the thyroid (25, 50, or 90%). These model predictions also include inhibition of NIS thyroidal iodide uptake by ClO_4_^−^. Data (mean ± SD) from Yu et al. (2002).

Figure 6A shows the 10-mg/kg/day dose group (Yu et al. 2002) model predictions for TT_4_ when a 25% (INH_p_ = 0.75) or 50% (INH_p_ = 0.50) inhibition of thyroid hormone synthesis is tested using the model. A 50% decrease in thyroid hormone production rate appears to describe the decreased TT_4_ concentrations for the first 5 days of exposure, but for serum TSH (Figure 6B) 90% inhibition of thyroid hormone synthesis did not describe the kinetic behavior for TSH. Serum TT_3_ was predicted fairly well without imposing inhibition of thyroid hormone production; however, a 25% decrease in thyroid hormone production provided a slightly better fit to the Yu et al. (2002) 10 mg/kg/day dose group [see Supplemental Material, Figure 2 (http://www.ehponline.org/members/2009/0800111/suppl.pdf)]. This simple simulation exercise appears to suggest that thyroid hormone production may be partially inhibited on a transient basis. However the relationship between TSH production and serum TT_4_ is complex and very different from dietary-iodide–induced hypothyroidism.

## Discussion

In this article, we report on the use of computational modeling analyses to examine the biological processes associated with disruption of an endocrine system, the HPT axis in the adult rat, by ClO_4_^−^. The BBDR-HPT/ClO_4_^−^ model simulation results suggest that ClO_4_^−^ administered in drinking water (or by other routes of administration) interacts with the rat thyroid gland itself, potentially altering thyroid hormone synthesis or secretion. This interaction is in addition to blocking thyroidal uptake of iodide. Current presumptions of a single MOA for ClO_4_^−^ on the HPT axis do not appear tenable based on these results.

Changes in serum TSH and TT_4_ after only 1 day of ClO_4_^−^ treatment were surprising because thyroidal iodide depletion was expected to be minimal over this time period, although 26–96% inhibition of NIS thyroidal iodide uptake was predicted by the model, which is in close agreement with Yu et al. (2002). The only reported effects of ClO_4_^−^ on thyroidal iodide content we found were those of Ortiz-Caro et al. (1983), who reported a decrease in thyroidal ^127^I of nearly 8% in rats administered 1,000 ppm ClO_4_^−^ in drinking water for 2 days and provided a low-iodide diet. A 10–15% decrease over 1 day is predicted by our model, if ClO_4_^−^ caused a 100% block of thyroidal uptake of dietary iodide. In comparison, for a severely iodide-deficient diet (0.3 μg I/day), it would take 7 days to deplete thyroid stores by 43% and result in a 35% decrease in serum T_4_ (McLanahan et al. 2008). This degree of perturbation in the HPT axis compares with the 10 mg/kg/day ClO_4_^−^ dose group after only 1 day of exposure (Yu et al. 2002). In the iodide-deficient BBDR-HPT axis model (McLanahan et al. 2008), the thyroidal iodide stores were closely related to iodide deficiency perturbations in the HPT axis. With ClO_4_^−^, it is apparent that the HPT axis is disturbed before sufficient model-predicted depletion of thyroidal iodide stores. For iodide deficiency, slow depletion of thyroidal iodide stores was governed by iodide available for thyroidal uptake and the secretion and metabolism rates of thyroid hormones.

McNabb et al. (2004a, 2004b) reported on the dose-dependent thyroidal depletion of T_4_ from bobwhite quail chicks ingesting ClO_4_^−^. This depletion in thyroidal T_4_ may have occurred because of the blocking effect of ClO_4_^−^ on thyroidal uptake of iodide, ClO_4_^−^ acting on the thyroid gland synthesis and secretion of T_4_, or a combination of both modes of action. The idea that ClO_4_^−^ may affect thyroid hormone synthesis/secretion has previously been discussed (Hildebrandt and Halmi 1981; Wolff 1998). Greer et al. (1966) provided experimental evidence for a direct effect on ClO_4_^−^ on the rat thyroid gland *in vitro*. These authors reported that ClO_4_^−^ reduced formation of MIT and DIT in rat thyroid lobes when incubated with ClO_4_^−^. They observed changes in MIT and DIT formation for ClO_4_^−^ media concentrations starting at 10 mg/L with a reported 50% effective reduction of DIT at 250 mg/L. By comparison, *in vivo*, rats ingesting 1, 3, and 10 mg/kg/day ClO_4_^−^ in drinking water had measured serum ClO_4_^−^ concentrations of approximately 0.3, 1.4, and 4.9 mg/L and thyroidal ClO_4_^−^ concentrations of 10, 50, and 176 mg/kg, respectively (Yu et al. 2002). Although difficult to compare, the *in vitro* media and *in vivo* serum ClO_4_^−^ concentrations suggest that the *in vitro* ClO_4_^−^-induced changes in organification may be a high-dose effect of ClO_4_^−^. Nevertheless, the data reported by Greer et al. (1966) demonstrate that interactions of ClO_4_^−^ and thyroid gland processes are possible.

Interestingly, Yu et al. (2002) also reported that when rats were administered an intravenous dose of 3 mg/kg of ClO_4_^−^, serum TSH and TT_4_ concentrations were at control levels 8 hr postdosing, but by 12 hr serum TSH concentrations increased and serum TT_4_ concentrations decreased. The secretion rate of iodide (as thyroid hormones) is about 0.5–1 μg over a 12-hr period, which suggests that thyroidal iodide stores may be depleted by only 5–8% when HPT axis perturbations were observed.

Männistö et al. (1979) administered propylthiouracil (PTU), MMI, potassium perchlorate (KClO_4_), and KI to rats in drinking water and measured serum TSH and TT_4_ at several time points over 14 days of treatment. KI and KClO_4_ both rapidly depleted serum TT_4_ concentrations by day 2 of treatment. KI animals recovered by day 4 of treatment, whereas the KClO_4_ rats did not, and serum TT_4_ concentrations remained low, similar to MMI and PTU animals, over 14 days of treatment. Excess iodide is well known for this Wolff-Chaikoff effect, which is associated with a transient “shutdown” in the thyroid gland (Wolff and Chaikoff 1948). MMI and PTU interact with thyroid peroxidase *in vitro*, but ClO_4_^−^ does not (Magnusson et al. 1984).

A recent *in vitro* study conducted by Tonacchera et al. (2004) in Chinese hamster ovary cells transfected with human NIS affirm that ClO_4_^−^ inhibits iodide uptake in a competitive fashion, with potencies 15, 30, and 240 times greater than the related compounds thiocyanate (SCN^−^), iodide, and nitrate (NO_3_^−^), respectively. The relative concentrations of these monovalent anions determine the ability of NIS to transport iodide, and because SCN^−^ and NO_3_^−^ are present in human sera at greater concentrations than ClO_4_^−^, several researchers (De Groef et al. 2006; Gibbs 2006) have suggested that SCN^−^ and NO_3_^−^ are more likely than ClO_4_^−^ to adversely affect (e.g., correlate with) thyroid function. However, Blount et al. (2006) reported correlations between ClO_4_^−^ and serum TT_4_ and TSH, no correlation with nitrate, but a negative association of TT_4_ with thiocyanate. That a decrease in serum TT_4_ and increase in serum TSH correlated with ClO_4_^−^ but not with the other monovalent anions suggests that ClO_4_^−^ may act through another MOA in addition to simple competitive inhibition.

TSH secretion from the pituitary is controlled by thyroid-releasing hormone (TRH) from the hypothalamus, and secretion of both TSH and TRH is partially controlled by thyroid hormones derived from serum. Using a quantitative evaluation of the pulsatile nature of the HPT axis, Li et al. (1995) suggest that the feedback effects of thyroid hormones on the hypothalamus and control of TRH secretion are much smaller than effects on the pituitary for control of TSH secretion. However, if TRH secretion is high, TRH may dominate the control of TSH secretion. In the case of ClO_4_^−^, the current BBDR–HPT axis model, calibrated for iodide deficiency, would need to be calibrated to explicitly include differential control of TSH secretion by both TRH and thyroid hormones.

Hypothalamic TRH does drive pituitary TSH secretion; however, details in rats, humans, and other species are not sufficient to distinguish between hypothalamic- and pituitary-derived TSH secretion. Measuring pituitary portal blood TRH is not realistic. A few attempts in rodents, using extreme surgical techniques, have been carried out, but not in reference to perturbations of the HPT axis. This aspect of HPT axis regulation remains a data gap. Eisenberg et al. (2008) recently published a control systems simulation model of the HPT axis feedback system in humans. They also did not include the hypothalamic aspect in their feedback simulator because of a lack of information. In our BBDR-HPT axis model (McLanahan et al. 2008), the TRH aspect of TSH production is embedded or lumped with the equation that describes the relationship between serum TSH and TT_4_ concentrations (Equation 3). Eisenberg et al. (2008) also lumped the hypothalamic contribution to describe TSH secretion in humans.

In conclusion, our computational analysis indicates that ClO_4_^−^ alters the HPT axis by blocking iodide transport and by interacting in some fashion with hormone production/release from the thyroid. The present description of synthesis and secretion of thyroid hormones (Equation 3) is simple, and if the thyroid gland itself is a target of ClO_4_^−^, as the modeling suggests, then a more complex description of the thyroid hormone synthesis and secretion process will be required. Our goal in this effort was to impose “nonmechanistic” outcomes to better understand what conditions may help explain the observed ClO_4_^−^ dose response. A significant correlation of effects of ClO_4_^−^ with serum thyroid hormones in humans has yet to be confirmed, so the importance of this potential MOA in humans is not known. Computational models can provide quantitative tools to evaluate possible MOAs of ClO_4_^−^ on the HPT axis. This evaluation would be supported by conducting specific studies to develop a quantitative mathematical description of the MOA, along with studies that measure temporal perturbations in several HPT axis end points. Studies are needed that evaluate the molecular biology of the genes within the thyroid gland, coupled with measurement of thyroid hormone precursors and thyroid hormones using modern analytical techniques such as liquid chromatography/mass spectrometry. These studies need to be completed for known iodine intake rates along with sufficient doses and time points to construct a temporal dose–response curve after ClO_4_^−^ exposure via drinking water.

## Figures and Tables

**Table 1:** Physiologic parameters for the adult rat perchlorate model.

Parameter	Value	Source
Tissue volumes (*V*)		
Plasma, *V_Pl_* (% BW)	4.44	Brown et al. 1997, Everett et al. 1956
Thyroid, *V_T_*_*_c_*_ (% BW)	0.005	McLanahan et al. 2007
Thyroid blood, *V_TB_*_*_c_*_ (% *V_T_*)	15.7	Malendowicz and Bednarek 1986
Rest of body, *V*_Body_ (L)	BW − *V_T_* − *V_Pl_*	
Blood flows (*Q*)		
Cardiac output, *Q_C_*_*_c_*_*_c_* (L/hr/kg^0.75^)	14.0	Brown et al. 1997
Thyroid, *Q_T_*_*_c_*_ (% *Q_C_*)	1.6^a^	Brown et al. 1997
Rest of body, *Q*_Body_	*Q_C_* − *Q_T_*	

BW, body weight; V_T_, volume of thyroid; Q_C_, cardiac output; Q_T_, blood flow to thyroid

a Human value.

**Table 2:** Compound-specific parameters.

Parameter	Value	Source
Partition coefficient (dimensionless)		
Body:blood, *PB_p_*	0.416^a^	Merrill et al. 2003
Permeability area cross-product (L/hr/kg^0.75^)		
Iodide, thyroid blood:thyroid tissue, *PAT_c_*_*_i_*_	1 × 10^−4^	McLanahan et al. 2008
ClO_4_^−^, thyroid blood:thyroid tissue, *PAT_c_*_*_p_*_	2.8 × 10^−4^	Merrill et al. 2003
Affinity constants (nmol/L)		
Iodide, thyroid NIS, *K_m_*_*_i_*_	3.15 × 10^4^	Merrill et al. 2003, Gluzman and Niepomniszcze 1983
ClO_4_^−^, thyroid NIS, *K_m_*_*_p_*_	1.5 × 10^3^	Kosugi et al. 1996
Maximum velocities (nmol/hr/kg^0.75^)		
Iodide, thyroid NIS, *V*_max_*T_c_*_*_i_*_	5.7 × 10^3^	McLanahan et al. 2008
ClO_4_^−^, thyroid NIS, *V*_max_*T_c_*_*_p_*_	1.8 × 10^2^	Optimized
Clearance (L/hr/kg^0.25^)		
Iodide, urinary excretion, *ClU_c_*_*_i_*_	5 × 10^−3^	McLanahan et al. 2008
ClO_4_^−^, urinary excretion, *ClU_c_*_*_p_*_	7.0 × 10^−2^	Optimized

a Weighted based on PBPK model partition coefficients. Data from Merrill et al. (2003).

**Table 3:** Model predictions of daily NIS uptake of iodide into thyroid (μg/day) and total thyroid iodide stores (μg) for a 300-g adult rat, after 1 and 14 days of ClO_4_^−^ exposure in drinking water at various doses.

	Dose (mg/kg/day)					
ClO_4_^−^ exposure	0	0.1	1	3	10	15
1 day						
Average amount of iodide transported by NIS into thyroid (μg/day)	38	28	10	4.6	1.7	1.2^a^
Total thyroid iodide stores (μg)	17.0	17.0	16.9	16.8	16.0	14.9^a^
14 days						
Average amount of iodide transported by NIS into thyroid (μg/day)	38	28	10	4.6	1.9	1.5
Total thyroid iodide stores (μg)	17.1	17.0	16.9	16.4	11.4	9.31

a Model simulation for 2 days of exposure to 15 mg/kg/day. Data from Männistö et al. (1979).
